# Dynamic Associations Between Public Risk Perception and Behavioral Responses on Weibo During the 2023 Beijing–Tianjin–Hebei Extreme Rainfall Event

**DOI:** 10.3390/bs16091510

**Published:** 2026-08-27

**Authors:** Yanyan Wang, Yumeng Fan

**Affiliations:** Faculty of Humanities and Social Sciences, Harbin Institute of Technology, Harbin 150001, China; wyy@hit.edu.cn

**Keywords:** natural disasters, risk perception, behavioral responses, Weibo, SOR model, 2023 Beijing–Tianjin–Hebei extreme rainfall event

## Abstract

Social media has become an important platform for the public to obtain information, express emotions, seek help, and participate in collaborative responses during major natural disasters. However, existing studies have mainly focused on the intensity of disaster-related discussions, emotional evolution, or patterns of information dissemination, while systematic examination remains limited regarding how public risk perception is associated with specific behavioral responses and whether these associations vary across disaster stages, degrees of regional impact, and user types. Using the stimulus–organism–response (SOR) framework as an organizing heuristic, this study examines the 2023 Beijing–Tianjin–Hebei extreme rainfall event. A total of 31,102 Sina Weibo posts were collected. By integrating LDA topic modeling, risk-perception dictionary matching, a BERT-based semantic robustness check, and chi-square tests, this study analyzes descriptive associations between public risk perception and behavioral responses. The results show that Weibo discussions evolved across disaster stages, with topics shifting from meteorological warnings and disaster reports to mutual aid, rescue operations, material support, and post-disaster reflection. Public risk perception shifted from information-oriented to relationship-oriented cognition, while behavioral responses transitioned from information attention to emergency mutual aid, sustained support, and reflective expression. Further analysis indicates that uncertainty perception was mainly associated with information attention; susceptibility perception co-occurred with help-seeking and emotional support; positive trust perception was strongly associated with emotional support; and negative trust perception co-occurred with questioning, accountability, and reflective suggestions. Because the negative-trust classifier performed substantially less well than the other dimensions, findings involving negative trust are treated as exploratory. Heterogeneity analyses show that observed perception–behavior associations varied across disaster stage, regional impact, and user type. These findings deepen understanding of social-media discourse during disasters while supporting cautious, stage-, region-, and group-sensitive risk communication.

## 1. Introduction

Climate change has increased the frequency and intensity of extreme weather events, making rainstorm-induced flooding an increasingly important threat to human safety, property, and urban resilience. In recent years, disasters have also become highly visible digital events. Social media platforms enable affected residents, government agencies, news organizations, rescue teams, and distant observers to exchange disaster-related information in real time. They have therefore become important spaces for warning dissemination, situational awareness, help-seeking, emotional expression, mutual aid, and public discussion during emergencies (Vieweg et al., 2010; Houston et al., 2015; Imran et al., 2015; Erokhin & Komendantova, 2024; Stolero et al., 2024). At the same time, the rapid circulation of fragmented, unverified, and emotionally charged information may intensify uncertainty and complicate disaster communication and emergency decision-making (Imran et al., 2015; Erokhin & Komendantova, 2024).

From late July to early August 2023, under the combined influence of the residual circulation of Typhoon Doksuri and the subtropical high, North China experienced its most severe rainfall event since 1963. Zhuozhou City in Hebei Province was particularly severely affected, with extensive inundation, infrastructure disruption, and serious threats to residents’ lives and property. The disaster generated sustained and large-scale discussion on Sina Weibo for more than 20 days. This case provides an opportunity to examine how the public perceived risk, responded to evolving disaster conditions, and participated in online collective action during a major rainstorm and flood event.

Risk perception is commonly understood as individuals’ subjective judgment of the likelihood and potential consequences of a hazard (Slovic, 1987). Existing theoretical frameworks have emphasized that public responses to risk are shaped not only by the objective characteristics of hazards but also by perceived severity, susceptibility, uncertainty, controllability, trust, and coping capacity. For example, the Health Belief Model conceptualizes threat appraisal through perceived severity and susceptibility (Rosenstock, 1974); the Protective Action Decision Model incorporates information sources, environmental cues, risk perception, protective action perception, and behavioral implementation into a decision-making process (Lindell & Perry, 2012); and Protection Motivation Theory highlights the joint roles of threat appraisal and coping appraisal (Rogers, 1975). In disaster contexts, these dimensions may be expressed through social media posts in which users describe disaster severity, concern about personal exposure, uncertainty regarding evolving conditions, or trust and distrust in authorities and rescue systems.

Public behavioral responses are similarly multidimensional. In addition to information seeking and information sharing, social media users may issue protective reminders, report local hazards, request assistance, provide resources, offer emotional support, express distress, demand accountability, or propose post-disaster improvements. Recent research has shown that social media can reveal public needs, emotional reactions, and risk-related behavior during disasters, while also highlighting challenges related to misinformation, uneven visibility of affected areas, and differences in information access across social groups and regions (Erokhin & Komendantova, 2024; He et al., 2023; Li et al., 2024; Ma et al., 2023; Zheng et al., 2023). Evidence also suggests that risk perception is associated with emergency information-seeking behavior, although this relationship may vary across emergency types, social contexts, and communication environments (Gong et al., 2026). Nevertheless, existing research has often examined disaster discussion intensity, topic evolution, emotional change, risk perception, or individual behavioral categories separately. Less attention has been paid to how different dimensions of risk perception are associated with specific forms of public behavioral responses and whether such associations change across disaster stages, regional impact levels, and user groups.

The stimulus–organism–response (SOR) framework provides a useful organizing perspective for addressing this gap. Originating in environmental psychology, the SOR model proposes that external stimuli influence internal psychological states, which subsequently shape behavioral responses (Mehrabian & Russell, 1974). In social-media-based disaster communication, warning messages, disaster reports, rescue information, and online narratives can be conceptualized as external stimuli; perceptions of severity, susceptibility, uncertainty, and trust represent organism-level appraisal; and information, protective, mutual-aid, and expressive behaviors constitute observable responses. In the present observational design, however, individual exposure to specific messages or hazard cues was not measured. Disaster stage is therefore used only as a contextual proxy for the changing information environment, not as a direct measure of stimulus exposure or a test of a complete causal SOR pathway. Cultural Theory further emphasizes that risk judgments are socially selected and shaped by shared values and institutional relations (Douglas & Wildavsky, 1982), while the Social Amplification of Risk Framework explains how information channels and social actors can amplify or attenuate risk signals (Kasperson et al., 1988). These perspectives qualify any mechanical or linear interpretation of SOR and help situate the observed associations within a broader cultural and communicative context.

Accordingly, this study takes the 2023 Beijing–Tianjin–Hebei extreme rainfall event as a case study and analyzes 31,102 Sina Weibo posts published between 27 July and 15 August 2023. By integrating time-series analysis, LDA topic modeling, multidimensional risk-perception dictionary matching, a BERT-based semantic robustness check, and chi-square tests, this study addresses three questions. First, how did public discussions, risk-perception indicators, and behavioral-response indicators vary across the warning, outbreak, rescue, and recovery stages? Second, how were different dimensions of textual risk perception associated with specific behavioral-response indicators? Third, how did these perception–behavior associations vary across disaster stages, degrees of regional impact, and user types?

This study contributes to the literature in three ways. First, it uses the SOR framework as a non-causal organizing heuristic for social-media-based disaster research and specifies an analytical pathway connecting the changing disaster-information context, multidimensional risk perception, and behavioral responses. Second, it combines interpretable dictionary-based identification with a BERT-based semantic robustness check while explicitly documenting the limitations of dictionary-derived labels and rare categories. Third, it describes dynamic and heterogeneous perception–behavior associations across stages, regions, and user groups, thereby providing evidence for stage-, region-, and group-sensitive disaster risk communication and public engagement strategies.

## 2. Materials and Methods

### 2.1. Case Selection and Data Sources

This study selected the 2023 Beijing–Tianjin–Hebei extreme rainfall event as the research case. The study period covered 20 days, from 27 July to 15 August 2023, and was divided into four stages: the warning stage (27–29 July), the outbreak stage (30 July–2 August), the rescue stage (3–8 August), and the recovery stage (9–15 August).

Sina Weibo text data were used as the main research object. To ensure the comprehensiveness and relevance of data collection, both core keywords and extended keywords were used. The keyword list is shown in Table 1.

### 2.2. Data Preprocessing

Because multiple keywords were used for data collection, the same Weibo post might have been retrieved multiple times if it matched different keywords. Therefore, the files corresponding to all keywords were merged into a complete dataset, and duplicate records were removed based on Weibo IDs. Invalid data were then excluded. The jieba word segmentation tool (version 0.42.1) was used, together with a customized disaster-domain dictionary, and 31,102 valid samples were ultimately obtained. To analyze the evolutionary characteristics of public risk perception and behavioral responses across different stages of the disaster event, the study period was divided into four stages based on the actual development process and key event nodes, as shown in Table 2.

### 2.3. Construction and Identification of the Risk Perception Dictionary

Based on the Health Belief Model and the psychometric paradigm of risk perception, public risk perception was operationalized in five dimensions: severity, susceptibility, uncertainty, positive trust, and negative trust. Severity refers to perceived consequences; susceptibility refers to perceived likelihood of being affected; uncertainty refers to confusion or anxiety associated with incomplete or conflicting information; and trust refers to evaluations of rescue capacity and information transparency. For each Weibo post, keyword matches were counted within each dimension. A post containing terms such as “serious” or “disaster,” for example, was coded as expressing severity perception, and the number of matched terms was used as a proxy for dimension intensity. The dictionary contained 311 entries: 77 severity, 75 susceptibility, 62 uncertainty, 42 positive-trust, and 55 negative-trust terms (Table 3). The dictionary was static and interpretable but could not reliably capture all colloquial, metaphorical, ironic, or context-dependent expressions. Its outputs should therefore be understood as operational indicators of textual risk discourse rather than direct measurements of an individual’s latent psychological state.

### 2.4. Measurement of Behavioral Responses

Based on the extended definition of behavioral responses in the Introduction, a multi-label coding system comprising four major categories and eleven subcategories was constructed. The major categories were information behavior, protective behavior, mutual-aid behavior, and expressive behavior. Each subcategory was defined by explicit inclusion and exclusion criteria and a corresponding set of keywords or regular-expression rules (Table 4). Text was normalized during preprocessing, and the Python (version 3.12.9) implementation applied every subcategory rule to each post; a post could receive more than one label when it satisfied multiple criteria. The full-dataset classification was therefore deterministic and rule-based rather than an independently trained machine-learning classifier. For independent validation, two human coders first completed a 100-post pilot used only to refine and freeze the codebook; they then independently coded a separate 900-post main sample while blind to the dictionary, rule-based, BERT, and each other’s outputs. The Main-900 combined simple random cases with targeted enrichment of rare and method-discordant cases. Each of the 16 labels was coded as present (1), absent (0), or unclear (9). Cohen’s kappa was calculated from paired binary judgments; unclear judgments and binary disagreements entered a separate codebook-based adjudication, and the agreed plus adjudicated labels formed the final validation reference. Across the eleven behavioral subcategories, raw agreement ranged from 93.3% to 100.0% and kappa from 0.688 to 1.000. Against the final Main-900 reference, the rule system had a macro precision of 0.513, a macro recall of 0.626, a macro F1 score of 0.519, and a micro F1 score of 0.550. Category-specific F1 values were 0.124 for information seeking, 0.759 for information forwarding, 0.694 for following official sources, 0.481 for advice and reminders, 0.546 for hazard reporting, 0.454 for help-seeking, 0.428 for offering help, 0.830 for emotional support, 0.485 for emotional venting, 0.708 for questioning/accountability, and 0.198 for reflective suggestions. Because the validation sample deliberately enriched rare and difficult cases, these unweighted class proportions and accuracy values should not be interpreted as population prevalence estimates.

### 2.5. Time-Series Analysis and LDA Topic Modeling

To analyze the temporal evolution of Weibo discussions, this study counted the number of Weibo posts by day, plotted a time-series curve, and marked key event nodes in the figure, including the release of meteorological warnings, typhoon landfall, flood peaks, and rescue progress.

To identify the core topics of public discussion, this study implemented Latent Dirichlet Allocation (LDA) using Gensim (version 4.4.0) on the preprocessed Weibo texts. Candidate solutions with 3–12 topics were inspected using c_v_ topic coherence together with semantic distinctiveness and interpretability across disaster stages. The highest recorded coherence in the archived model-selection output was 0.6395 at three topics; however, that solution merged substantively distinct warning, rescue, mutual-aid, and recovery discussions. The six-topic solution was retained as a theory-informed descriptive partition because it separated these event functions without producing highly redundant labels. Coherence was therefore used as a diagnostic rather than as a single automatic maximization rule. Topics were named by comparing high-probability terms with manually reviewed example posts. Dynamic topic models can represent gradual temporal drift (Blei & Lafferty, 2006), non-negative matrix factorization offers an alternative factorization of term-document matrices (Lee & Seung, 1999), biterm topic models are designed for sparse short texts (Yan et al., 2013), and BERTopic uses contextual embeddings and class-based TF–IDF (Grootendorst, 2022). LDA was retained here for its transparent word distributions and compatibility with stage-wise descriptive comparisons; the absence of a formal cross-model robustness comparison remains a limitation. The original LDA formulation is described by Blei et al. (2003). A frozen-data reproducibility rerun used 31,102 rows (30,937 non-empty documents), a 10,692-term vocabulary, random seed 42, and 10 passes. For k = 3–12, the c_v_ coherence values were 0.6395, 0.5635, 0.5843, 0.5649, 0.5695, 0.5551, 0.6096, 0.5395, 0.5629, and 0.5108, while perplexity values were 186.92, 169.37, 163.13, 162.26, 163.89, 159.38, 157.36, 164.34, 182.55, and 215.53, respectively. Thus, k = 3 maximized coherence and k = 9 minimized perplexity; neither diagnostic alone selected k = 6. The six-topic solution was retained by an explicit multi-criterion rule: it formed a local perplexity minimum relative to k = 5 and k = 7, preserved distinct warning, rescue, material-support, help-seeking, loss/compensation, and commemorative functions, and avoided the functional merging at k = 3 and the fragmentation and narrow event-specific clusters at k = 9. The retained solution should therefore be interpreted as a parsimonious and substantively useful partition rather than the numerical optimum of a single metric.

### 2.6. BERT-Based Semantic Robustness Check

To examine whether a contextual language model could reproduce and extend the dictionary-derived labels, this study fine-tuned bert-base-chinese on 2000 posts sampled from the dictionary-labeled corpus. The label counts were 1129 for severity, 751 for susceptibility, 389 for uncertainty, 682 for positive trust, and 109 for negative trust; labels were not mutually exclusive. The archived implementation used an 80/20 training–validation split (1600/400 posts), a sigmoid output for each label, and a 0.50 decision threshold. Precision, recall, and F1 score were first calculated against the held-out dictionary-derived labels. The macro-average F1 score across the five dimensions was 0.740, but performance was highly uneven, especially for the rare negative-trust category. Because this internal reference inherited the dictionary, BERT was treated as a semantic robustness and error-discovery model rather than as independent validation. Independent construct validation used the blind Main-900 reference described above. Across the five risk-perception dimensions, raw inter-coder agreement ranged from 92.3% to 100.0% and Cohen’s kappa from 0.450 to 1.000 (mean kappa = 0.784); the low kappa for negative trust was interpreted together with its 95.7% raw agreement and very low positive prevalence. Against the final Main-900 reference, dictionary and BERT macro F1 scores were 0.517 and 0.492, respectively, while their micro F1 scores were 0.632 and 0.622. The underlying BERT architecture follows Devlin et al. (2019).

### 2.7. Statistical Analysis

To examine bivariate associations between risk perception and behavioral responses, this study constructed a “risk-perception dimension × behavioral-response type” cross-analysis matrix and used chi-square tests to assess departures from independence. Percentage-point differences were calculated as the behavioral occurrence rate among posts with a given perception minus the rate among posts without it. Heterogeneity analyses compared these descriptive associations across disaster stage, regional impact, and user type. These tests do not adjust for confounding, repeated observations, differential posting activity, or the endogeneity of perceptions and behaviors expressed in the same text. Accordingly, the results are interpreted as descriptive co-occurrence patterns rather than causal effects; adjusted multivariable modeling is identified as a necessary extension. We accept the value of adjusted modeling; however, the archived data do not contain a pre-specified and sufficiently validated set of individual-level confounders, and key variables such as demographics, verified exposure, and residence are unavailable. A post hoc logistic model based only on text-derived indicators could therefore create false precision, so no additional multivariable model was fitted in this revision. Thus, the present chi-square analyses and percentage-point differences address an exploratory bivariate question, whereas pre-specified multivariable or multilevel logistic models are required in future research to estimate adjusted associations.

## 3. Results and Analysis

### 3.1. Social Media Discussions and Topic Evolution

#### 3.1.1. Temporal Evolution of Discussions

**Figure 1:**
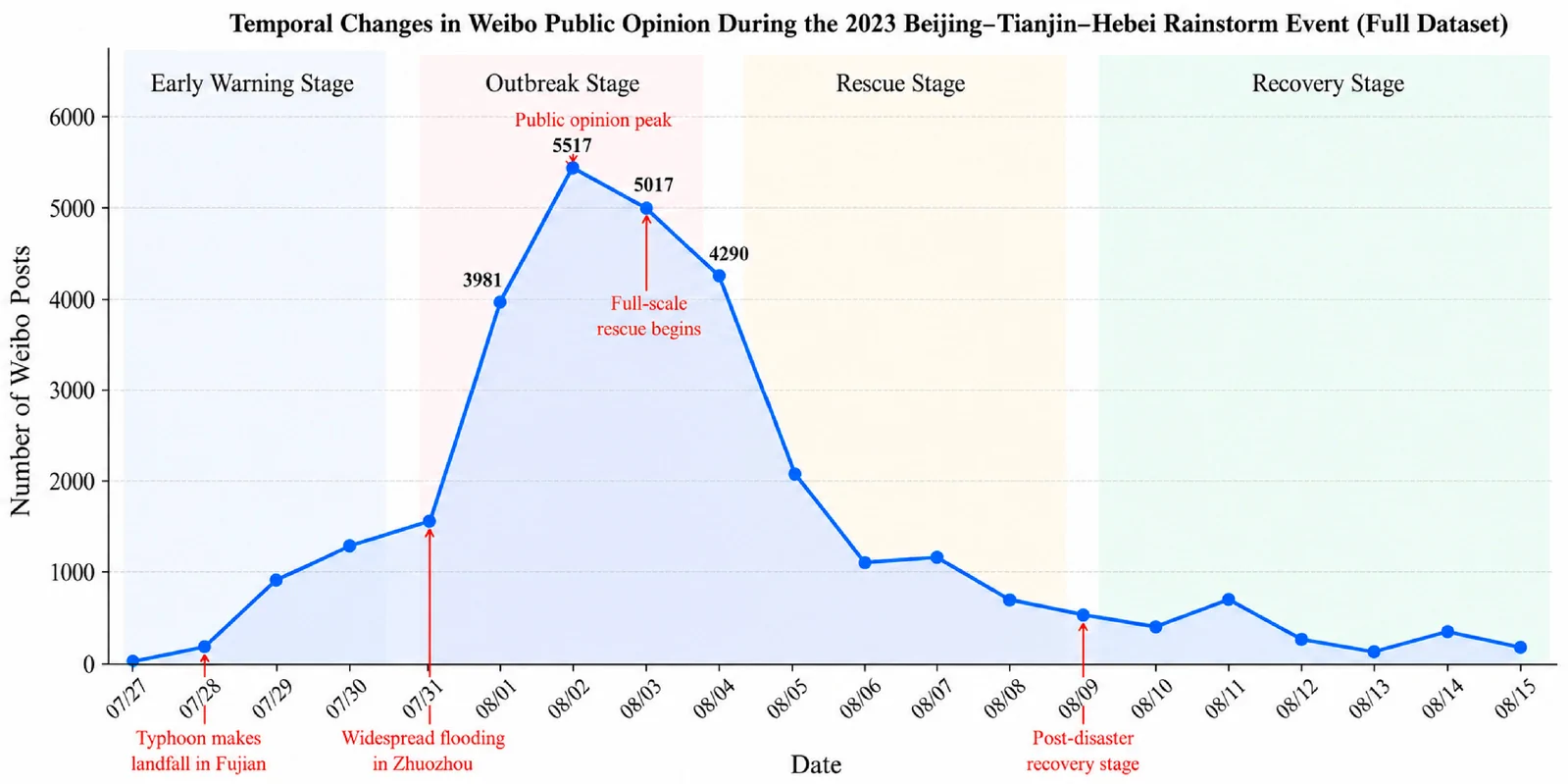
Temporal changes in Weibo discussions during the 2023 Beijing–Tianjin–Hebei extreme rainfall event.

After the 31,102 valid Weibo posts were counted by date, the results showed that Weibo discussions during the “23·7” extreme rainstorm in North China exhibited clear stage-based fluctuations, following an overall inverted U-shaped pattern of “slow increase–rapid rise–high-level fluctuation–gradual decline” (see Figure 1). This pattern was generally consistent with the actual development of the disaster, indicating that the intensity of Weibo discussion could effectively reflect the public’s dynamic attention to the disaster event.

During the warning stage, the volume of public discussion was relatively low, mainly focusing on the typhoon path, rainstorm warnings, travel safety, and risk reminders. After the event entered the outbreak stage, as heavy rainfall intensified and severe flooding occurred in Zhuozhou and other areas, the number of Weibo posts increased rapidly, with a marked rise in content related to help-seeking, trapped residents, waterlogging, and rescue. During the rescue stage, discussions remained at a high level, and public attention gradually shifted from the disaster itself to rescue progress, material support, and social mutual aid. In the recovery stage, as the disaster situation gradually stabilized, discussion intensity declined overall, while topics such as post-disaster cleanup, loss assessment, responsibility discussion, and recovery and reconstruction began to increase.

**Figure 2:**
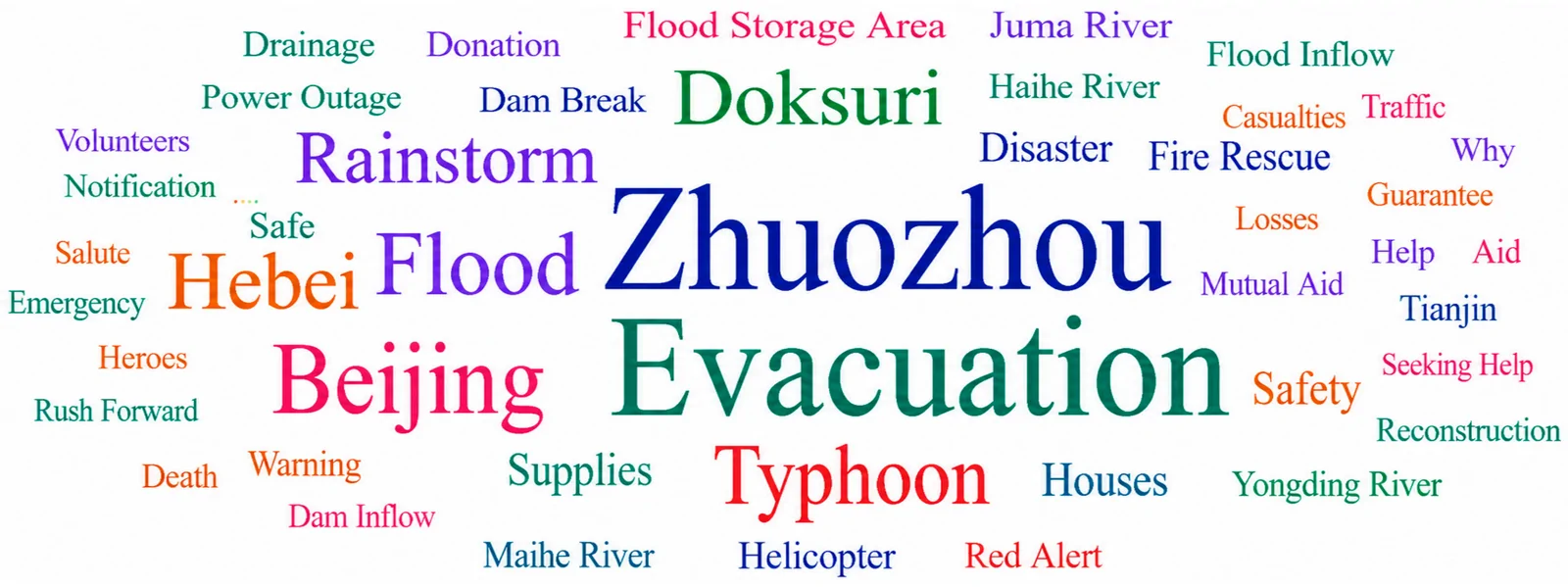
Keyword word cloud of Weibo texts related to the 2023 Beijing–Tianjin–Hebei extreme rainfall event.

To further present the overall characteristics of public discussion, this study generated a keyword word cloud based on the preprocessed Weibo texts (see Figure 2). The results showed that words such as “Zhuozhou,” “rescue,” “rainstorm,” “flood,” “help-seeking,” “firefighters,” and “materials” appeared frequently, indicating that public discussion was mainly concentrated on disaster information, rescue operations, mutual aid and help-seeking, and social support.

#### 3.1.2. Results of LDA Topic Modeling

This study applied LDA topic modeling to 31,102 valid Weibo texts. As described in Section 2.5, the six-topic solution was selected as an interpretable descriptive partition after examining candidate solutions with 3–12 topics; the highest archived c_v_ coherence value was 0.6395 at three topics, but that solution merged substantively distinct disaster-response functions. The retained model used a vocabulary of 10,692 terms. As shown in Table 5, the six topics concerned property loss and compensation claims; rescue operations; government disaster relief and material support; meteorological warnings and disaster reports; help-seeking, mutual aid, and on-site disaster records; and tributes to heroes and touching stories. In the frozen-data diagnostic rerun, k = 6 had c_v_ = 0.5649 and perplexity = 162.26, compared with c_v_ = 0.6395 and perplexity = 186.92 at k = 3 and c_v_ = 0.6096 and perplexity = 157.36 at k = 9. This confirms that the reported six-topic structure reflects the stated balance of semantic granularity, parsimony, and diagnostic fit rather than simple maximization of coherence or minimization of perplexity.

#### 3.1.3. Stage-Based Evolution of Topics

A further comparison of topic distributions across the four disaster stages showed that the focus of public discussion shifted markedly as the disaster progressed (see Table 6). During the warning stage, “meteorological warnings and disaster reports” was the dominant topic, accounting for 82.0% of all topics, indicating that before the disaster occurred, the public mainly relied on Weibo to obtain meteorological information and risk alerts. After the event entered the outbreak stage, the topic of “mutual aid, help-seeking, and disaster records” increased rapidly, accounting for 24.9% and becoming one of the most prominent issues at this stage. This suggests that once the disaster impact shifted from a potential risk to actual damage, public attention quickly turned to trapped residents, help-seeking, and on-site disaster conditions.

During the rescue stage, the proportions of the topics “rescue operations” and “government disaster relief and material support” increased significantly, reaching 24.8% and 21.2%, respectively. This indicates that, with the continued involvement of professional rescue forces and social resources, public attention shifted from the disaster impact itself to rescue progress and material support. In the recovery stage, the proportion of “government disaster relief and material support” further increased to 33.6%, while “property loss and compensation claims” remained at a relatively high level, suggesting that public discussion gradually shifted toward post-disaster recovery, loss assessment, and subsequent governance.

### 3.2. Evolution of Risk Perception

#### 3.2.1. Stage-Based Evolution of Risk Perception

**Figure 3:**
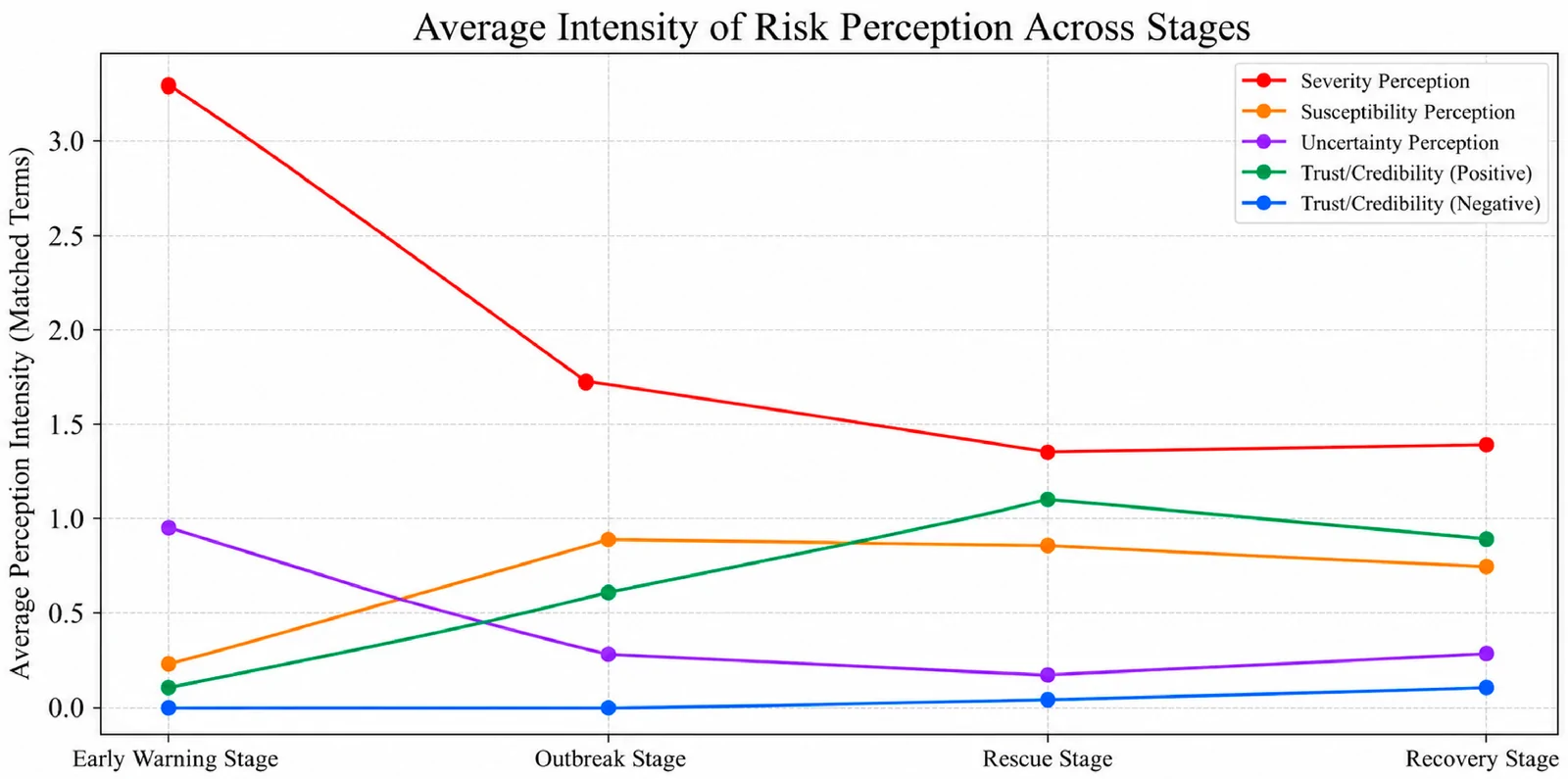
Stage-based changes in hit rates across risk perception dimensions.

Based on dictionary matching, textual indicators of severity, susceptibility, uncertainty, positive trust, and negative trust exhibited different frequencies across the four disaster stages (Table 7 and Figure 3).

Severity-related terms remained frequent throughout the study period but showed an overall downward trend. During the warning stage, 87.3% of posts matched the severity dictionary, indicating that language describing serious rainfall and flood consequences was already prominent before the disaster’s main impact. The corresponding rates declined to 61.6%, 51.1%, and 53.8% during outbreak, rescue, and recovery. This textual pattern suggests that explicit severity language became less dominant as discussion diversified toward exposure, rescue, support, and recovery.

Susceptibility-related language first increased and then decreased. Its hit rate was 15.4% during warning, rose to 41.2% during outbreak, remained at 39.4% during rescue, and declined to 30.6% during recovery. The increase coincided with posts about people being trapped, roads being waterlogged, and property damage. Because the dictionary does not identify the poster’s actual exposure or residence, this pattern indicates greater textual attention to being affected rather than a measured increase in individual susceptibility perception.

Uncertainty-related language showed an overall downward trend. Its hit rate was 56.4% during warning, compared with 22.8% during outbreak, 14.8% during rescue, and 17.3% during recovery. Early posts frequently referred to unclear typhoon paths, rainfall intensity, flood extent, and flood-control arrangements. The decline coincided with additional official releases and media coverage, although the observational data cannot determine whether those communications caused individual uncertainty to fall.

Positive-trust language increased and then declined slightly. Its hit rate was 8.7% during warning, 26.4% during outbreak, 42.2% during rescue, and 37.2% during recovery. Posts during rescue frequently praised firefighters, armed police, the People’s Liberation Army, social rescue teams, volunteers, and other actors. This co-occurrence is consistent with favorable evaluation of rescue and mutual-aid networks, but it does not establish that observed rescue actions caused individual trust to increase.

Dictionary-coded negative-trust language remained infrequent but increased from 1.8% during warning to 3.1% during outbreak, 6.9% during rescue, and 9.3% during recovery. Later posts more often discussed the adequacy of warnings, drainage systems, and institutional responses. Because negative trust was rare and the BERT classifier performed poorly for this category, the apparent increase is treated as an exploratory textual pattern rather than a precise estimate of public distrust.

#### 3.2.2. BERT Semantic Robustness Check

**Figure 4:**
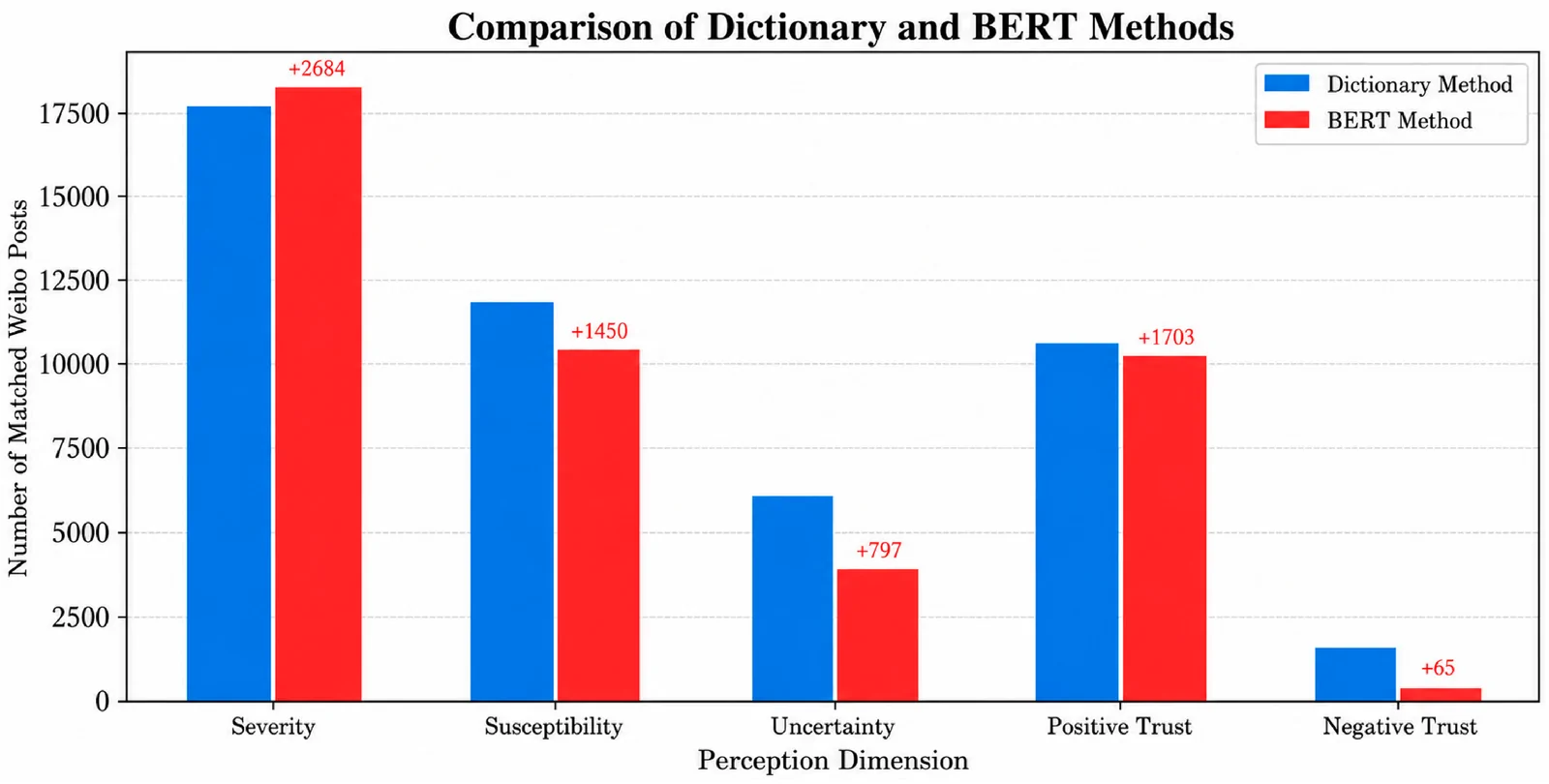
Visual comparison between the dictionary-based method and the BERT-based method.

A BERT-based model trained on the dictionary-derived labels was applied to the full dataset, and its outputs were compared with the original dictionary matches (Table 8 and Table 9; Figure 4). The new independent Main-900 validation showed that model–dictionary agreement did not by itself establish construct validity. Against the final reference, dictionary F1 scores for severity, susceptibility, uncertainty, positive trust, and negative trust were 0.861, 0.638, 0.439, 0.416, 0.229; the corresponding BERT F1 scores were 0.845, 0.548, 0.347, 0.371, 0.349. Thus, both methods captured severity comparatively well but produced many false positives for the trust and uncertainty dimensions. The original dictionary–BERT comparison is retained as a sensitivity analysis, while substantive claims are now calibrated to the independent validation results.

For severity perception, BERT additionally identified 2684 posts not covered by the dictionary; inspection of sampled cases suggested that many used colloquial or implicit expressions. Model–dictionary overlap exceeded 80% for severity, susceptibility, and positive trust, but the independent Main-900 results showed that overlap was not equivalent to accuracy. Negative trust was the weakest risk-perception dimension. Coders agreed on 95.7% of comparable cases, but Cohen’s kappa was 0.450 because positive judgments were rare and unevenly distributed (17 positives for coder A and 56 for coder B among the comparable main cases). After codebook-based adjudication, 45 posts were positive. The dictionary achieved a precision of 0.133, a recall of 0.822, an F1 score of 0.229, and an accuracy of 0.723; BERT achieved a precision of 0.220, a recall of 0.844, an F1 score of 0.349, and an accuracy of 0.842. Representative false positives included general anger or criticism without a clearly identified trust target, rhetorical questions, and news reports that mentioned institutional failure without endorsing the claim. Negative-trust estimates and related associations therefore remain exploratory.

### 3.3. Evolution of Behavioral Responses

#### 3.3.1. Overall Distribution of Behavioral Responses

Using the deterministic multi-label rule system described in Section 2.4, this study identified behavioral-response indicators in the full set of Weibo texts. The overall distribution is shown in Table 10. Mutual-aid indicators were most frequent during the “23·7” extreme rainstorm in North China (43.1%), followed by protective (24.8%) and information indicators (23.5%); expressive indicators accounted for 8.6%. These percentages describe coded textual signals and should not be interpreted as validated measures of offline behavior. Given the comparatively lower validation performance of information seeking (F1 = 0.124) and reflective suggestions (F1 = 0.198), the corresponding percentage patterns in Section 3.3 are treated as exploratory and interpreted with appropriate caution.

Within information behavior, references to official accounts were relatively frequent, showing the visibility of official channels in disaster and rescue posts. Suggestions and reminders and hazard-reporting indicators occurred at similar rates within protective behavior. Help-seeking and emotional-support indicators were the most frequent mutual-aid subcategories. These patterns show how Weibo was used to post requests, relay hazard information, and express support; they do not verify that users received information from the cited source or that help posted online produced an offline response.

#### 3.3.2. Stage-Based Evolution of Behavioral Responses

**Figure 5:**
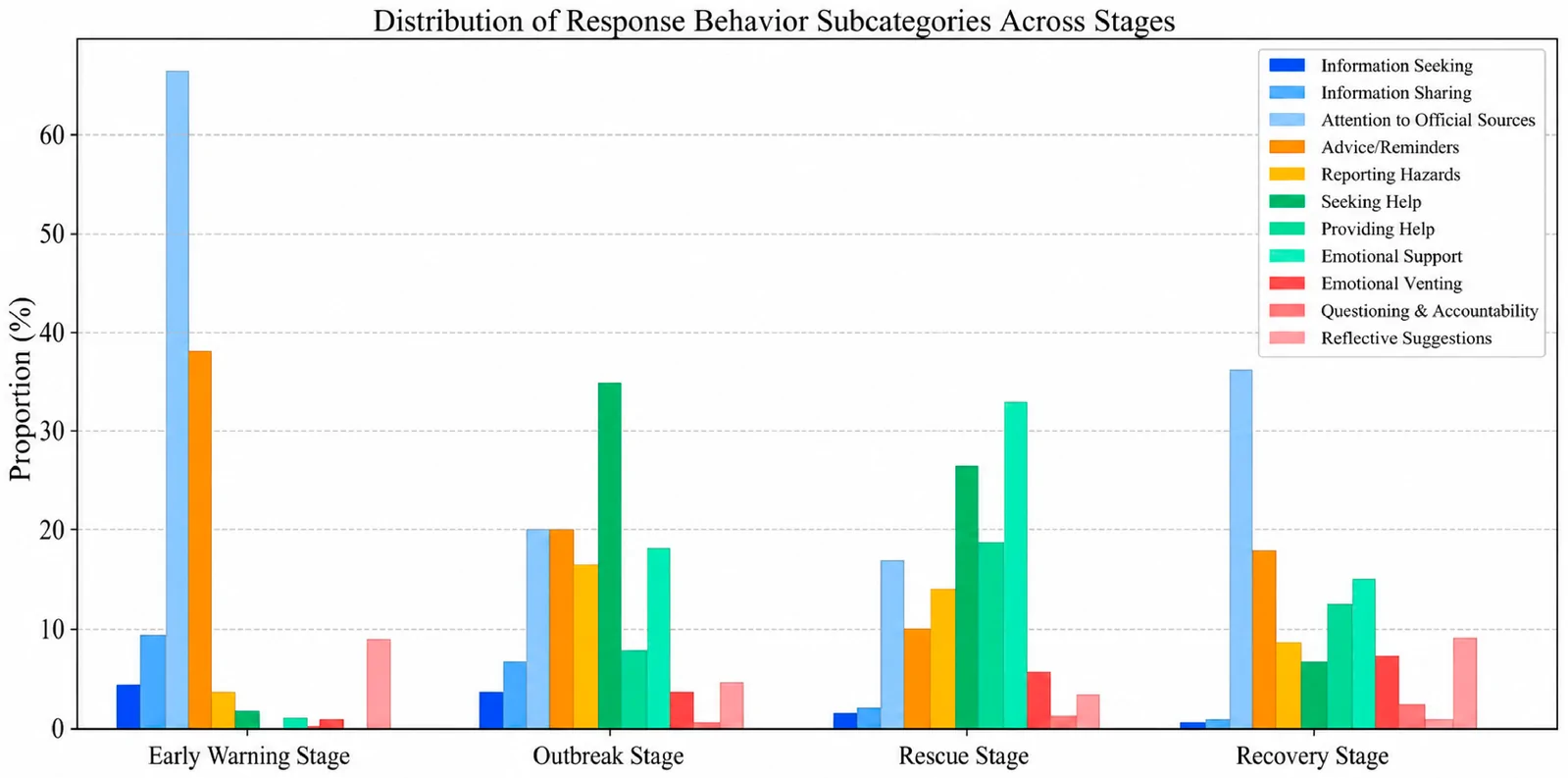
Distribution of behavioral responses subcategories across stages.

The four major categories of behavioral responses exhibited significant structural changes across the four stages, revealing a systematic shift in the focus of public behavior as the disaster progressed. The specific hit-rate data are shown in Table 11, and the stage-based trends of behavioral subcategories are presented in Figure 5.

During the warning stage, information indicators were most frequent (69.6%), followed by protective indicators (39.3%), while mutual-aid indicators remained low (2.6%). Users mainly discussed meteorological warnings, reposted official information, and offered safety advice. During the outbreak stage, mutual-aid indicators rose to 48.6%, while information indicators fell to 27.5%; help-seeking posts and online coordination became more visible as flooding worsened. During the rescue stage, mutual-aid indicators peaked at 55.8%, with increased provision-of-help and emotional-support signals. These online expressions may have supported awareness and coordination, but the dataset does not establish whether they produced completed offline rescue or material assistance. In the recovery stage, mutual-aid indicators fell to 24.6%, while expressive indicators rose to 16.8%; questioning, accountability, and reflective suggestions reached their stage peaks. The sequence is therefore interpreted as a change in online discourse from immediate information needs toward coordination, support, and later evaluation, rather than as a verified progression of offline behavior.

Across the eleven coded subcategories, official-account references and suggestions or reminders were most frequent during the warning stage; help-seeking indicators peaked at 34.8% during the outbreak stage; and offers of help and emotional-support indicators increased later. Questioning and accountability remained infrequent but rose from nearly zero during the warning stage to 2.4% during the recovery stage. This pattern is consistent with a shift in online expression from immediate information and assistance toward later evaluation, while not establishing a fixed public behavioral sequence.

#### 3.3.3. Behavioral Differences Across User Groups

Users were divided into two groups, official accounts and ordinary users, to compare behavioral differences between the two groups. The results are shown in Table 12.

### 3.4. Association Analysis Between Risk Perception and Behavioral Responses

**Figure 6:**
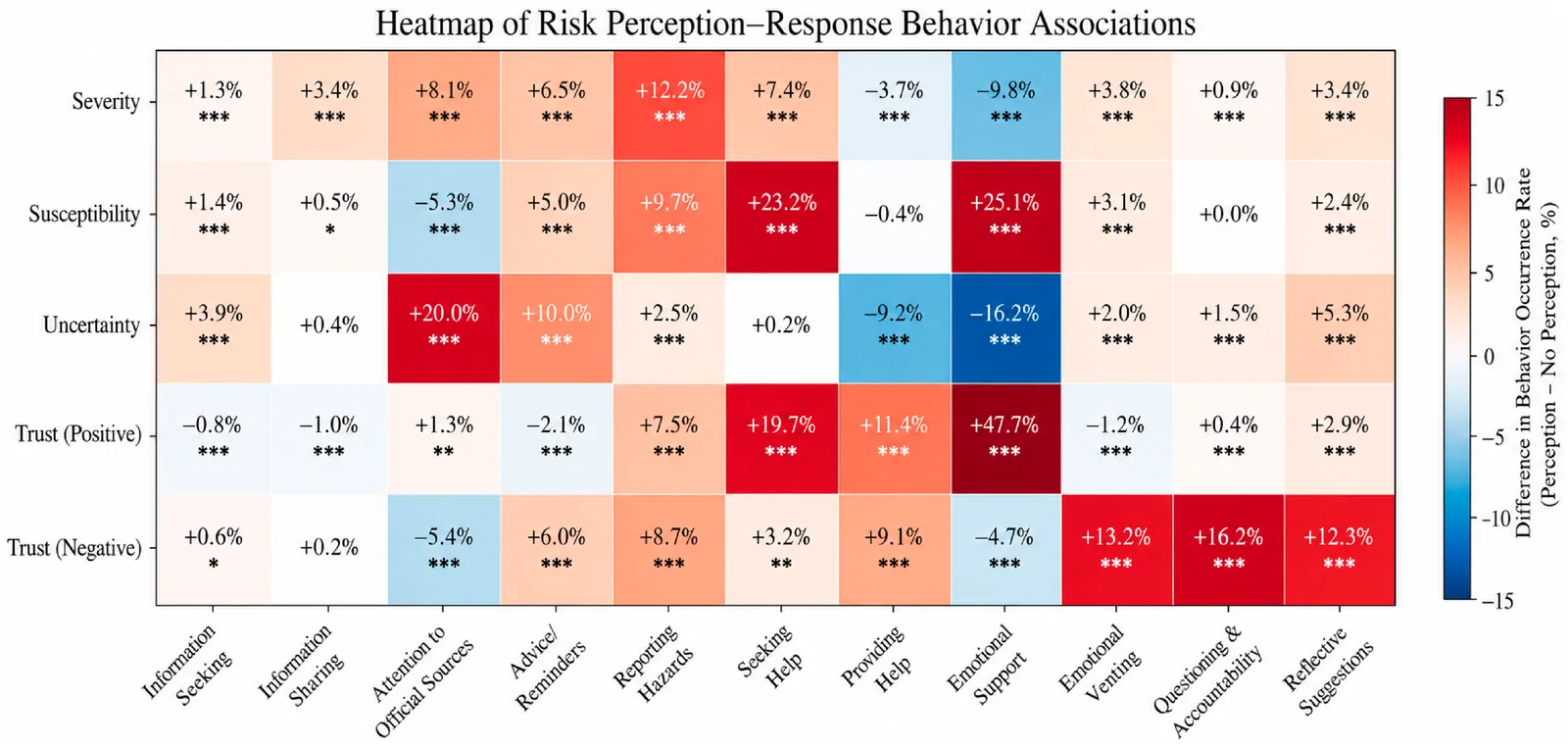
Heatmap of unadjusted associations between risk perception and behavioral responses. Note: Cells involving negative trust in this heatmap represent exploratory textual co-occurrences. In the Main-900 validation, this dimension had low positive prevalence, a precision of 0.133 for the dictionary and 0.220 for BERT, and a prevalence-sensitive Cohen’s kappa of 0.450; it should not be read as a validated measure of public institutional distrust. Statistical significance is denoted by * *p* < 0.05, ** *p* < 0.01, and *** *p* < 0.001. Black and white asterisks have the same statistical meaning; their color varies only to maintain visibility against different cell backgrounds.

To examine bivariate associations between risk-perception dimensions and behavioral-response indicators, this study constructed a 5 × 11 perception–behavior matrix. Chi-square tests assessed departures from independence, and percentage-point differences summarized the direction and magnitude of each unadjusted association. These statistics do not measure promoting or inhibiting effects. The results are shown in Table 13, and Figure 6 presents the corresponding heatmap. Likewise, associations involving information seeking and reflective suggestions are treated as exploratory and interpreted cautiously in light of their comparatively lower validation performance (F1 = 0.124 and 0.198, respectively).

Risk-perception and behavioral-response indicators exhibited distinct bivariate co-occurrence patterns. Positive trust co-occurred with emotional support; susceptibility was associated with help-seeking and emotional support; uncertainty was associated with information behavior; and severity was associated with information and protective behaviors. Negative-trust indicators also co-occurred with expressive behaviors, but this result is exploratory because of the weak negative-trust classification performance and low category prevalence.

### 3.5. Multidimensional Heterogeneity Analysis of Perception–Behavior Associations

#### 3.5.1. Stage-Based Dynamics of Perception–Behavior Associations

The four major categories of behavioral responses exhibited significant structural changes across the four stages, revealing a systematic shift in the focus of public behavior as the disaster progressed. The specific hit-rate data are shown in Table 14.

Table 14 shows that the largest unadjusted perception–behavior differences varied by stage. During the warning stage, negative trust and reflective suggestions displayed a large percentage-point difference; however, the estimate is based on a rare and weakly classified category and should not be given substantive weight without independent validation. During the outbreak and rescue stages, positive trust and emotional support formed the largest observed association. During recovery, negative trust co-occurred with reflective suggestions and emotional venting, but these estimates remain exploratory for the same measurement reasons. The changing pattern is consistent with context-sensitive appraisal, yet it does not demonstrate a directional or causal SOR transmission process.

#### 3.5.2. Regional Differences in Perception–Behavior Associations

Based on the geographic information mentioned in Weibo texts, the data were divided into three categories: severely affected areas, including Zhuozhou, Mentougou, and Fangshan (*N* = 20,250); generally affected areas (*N* = 4913); and unaffected or unspecified areas (*N* = 5939).

As shown in Table 15, susceptibility–help-seeking and positive trust–emotional support associations decreased from severely affected to unaffected or unspecified areas, a pattern consistent with an experiential-distance interpretation. Uncertainty–attention to official accounts showed the opposite gradient. These differences may reflect variation in direct experience, information access, posting practices, and the mix of users captured in each location category. The stronger negative trust–questioning association in severely affected areas is treated as exploratory because negative-trust measurement was weak. Similar susceptibility–emotional support differences across regions should not be interpreted as evidence of a universal cognitive mechanism.

#### 3.5.3. Differences in Perception–Behavior Associations by User Type

According to Table 16, the observed directions of association were similar across official and ordinary accounts. This descriptive consistency does not establish a common causal mechanism, particularly because account categories differ in institutional role, audience, posting frequency, and communication objectives.

#### 3.5.4. Stage × Region Interaction Analysis

Table 17 and Table 18 show a descriptive pattern of earlier response indicators in severely affected areas and later diffusion in more distant areas. The positive trust–emotional support association peaked during the rescue stage in all region categories and was largest in generally affected areas. One possible interpretation is that users in those areas combined some disaster experience with greater capacity to post supportive messages, but this explanation cannot be distinguished from differences in platform participation, account composition, or geographic classification.

## 4. Discussion

Using the 2023 Beijing–Tianjin–Hebei extreme rainfall event as a case study, this study examined dynamic associations between public risk-perception and behavioral-response indicators in 31,102 Weibo posts. By combining LDA topic modeling, multidimensional dictionary identification, a BERT-based semantic robustness check, and chi-square tests, the study showed that online disaster communication changed in content as well as volume. The findings are descriptive patterns in platform discourse; they are not estimates of individual psychological change or causal behavioral effects.

First, the findings revealed a clear stage-based restructuring of public discussion. During the warning stage, Weibo posts were mainly oriented toward meteorological information, disaster alerts, and precautionary reminders. Once flooding became widespread, the discussion rapidly shifted toward help-seeking, trapped residents, rescue operations, and mutual aid. During the rescue stage, public attention increasingly focused on rescue progress, material support, and emotional encouragement. In the recovery stage, discussions became more reflective, with greater attention to loss assessment, accountability, and institutional improvement. This pattern suggests that social media discussion during disasters follows the practical demands generated by changing disaster conditions. Public attention does not simply rise and fall with the severity of an event; it is redirected as the dominant problems faced by affected communities change over time.

Second, risk-perception indicators shifted from information-oriented toward relationship-oriented content. Severity and uncertainty were prominent during the warning stage; susceptibility increased as flooding produced visible impacts; and positive trust was most frequent during rescue. Dictionary-coded negative trust increased during recovery, when discussion turned toward warnings, infrastructure, and institutional responsibility. This last pattern must be interpreted cautiously: in the independent Main-900 validation, negative trust had low positive prevalence, a Cohen’s kappa of 0.450, and low precision for both the dictionary (0.133) and BERT (0.220), indicating a substantial false-positive risk from irony, general venting, criticism, and news reporting. The data nevertheless support the broader conclusion that the content of online risk discourse varied with the material and social context of the disaster.

Third, behavioral-response indicators shifted from information attention toward online mutual aid, support, and reflective expression. Information indicators dominated the warning stage, whereas help-seeking, offers of help, and emotional support became more visible during outbreak and rescue. Social media can serve as an informal coordination space in which needs and offers are posted, but a post does not show whether resources were verified, delivered, or converted into effective offline mitigation. This online–offline gap is consistent with the risk-perception paradox: high perceived risk or active communication does not necessarily lead to protective action (Wachinger et al., 2013). The observed sequence should therefore be understood as a change in online expression, not as direct evidence of resilience outcomes.

Fourth, different risk-perception indicators were associated with distinct behavioral-response indicators. Uncertainty co-occurred with attention to official sources and reminders, susceptibility with help-seeking and emotional support, and positive trust with emotional support, especially during rescue. These patterns may reflect differences in information needs and relational appraisal, but direction cannot be inferred because perception and behavior were coded from the same posts. Associations involving negative trust and expressive categories are particularly uncertain because both measurement reliability and prevalence were low. The analysis therefore identifies hypotheses about possible behavioral pathways rather than demonstrating that one form of cognition activated another response.

The heterogeneity analysis showed that bivariate perception–behavior associations varied by disaster stage, regional classification, and account type. This pattern is consistent with a differentiated communication ecology, but it also reflects the limits of the sample. Weibo users are not representative of the affected population: platform participation varies by age, socioeconomic status, digital access, political expression, institutional affiliation, and posting intensity. Geographic labels were inferred from text mentions rather than verified residence or exposure, and official accounts have different communication mandates from ordinary users. These selection and measurement processes may contribute to the apparent regional and user-group differences.

Methodologically, dictionary matching and contextual language models offer complementary but limited evidence. The dictionary is transparent and auditable, whereas BERT can flag colloquial or implicit expressions that static keywords miss. However, the BERT model was trained on dictionary-derived labels, so agreement cannot establish construct validity, and poor negative-trust performance shows that semantic sensitivity was uneven. Human interpretation remains essential for distinguishing irony, metaphor, distress, criticism, and distrust. The combined approach is therefore best treated as an exploratory large-scale text-measurement strategy that requires independent annotation and sensitivity analysis before strong substantive claims are made.

## 5. Conclusions

This study analyzed dynamic associations between risk-perception and behavioral-response indicators in disaster-related Weibo discourse. During the “23·7” extreme rainstorm in North China, discussion shifted from meteorological information and warnings toward rescue, mutual-aid, support, and post-disaster reflection. Risk-perception content moved from information-oriented toward relationship-oriented themes, while coded behavioral-response signals changed from information attention toward online mutual aid, support, and reflective expression. Different perception dimensions displayed distinct unadjusted associations with behavioral indicators, and these patterns varied across disaster stage, regional classification, and account type. The observational text design does not establish causal effects or verified offline behavior.

The findings support formal, non-discretionary workflows for using social-media signals in emergency governance. Agencies should define an auditable sequence of intake, automated triage, duplicate detection, human verification, priority assignment, routing to the responsible unit, response logging, closure, and post-event review. No help-seeking post should be routed directly to field operations solely on the basis of an automated label; location, urgency, identity, and duplication require verification, and personally identifiable information should be protected. During warning, agencies should issue stable and actionable meteorological and protective guidance while documenting update intervals and uncertainty. During outbreak and rescue, verified requests and offers can supplement—not replace—established dispatch and community reporting channels. During recovery, agencies should publish response records, correction mechanisms, accountability findings, and lessons learned through predefined procedures rather than relying on managers’ subjective interpretation of online sentiment. A two-layer system combining transparent keyword alerts with semantic review may assist triage, but it requires calibrated thresholds, human oversight, bias monitoring, and periodic external audits.

This study has several limitations. First, it examined one rainstorm–flood event on Sina Weibo. Users, posts, and geographic mentions are not representative of the affected population, and demographic, regional, institutional, and political posting biases limit generalizability. Second, independent blind coding improved the measurement audit but did not eliminate construct uncertainty. Inter-coder agreement was generally high, yet negative trust had a prevalence-sensitive kappa of 0.450, and dictionary/BERT validation showed low precision for uncertainty and trust. Third, behavioral-rule performance varied substantially: emotional support, information forwarding, following official sources, and questioning/accountability performed comparatively well, whereas information seeking, reflective suggestions, advice, help-seeking, and offering help were weaker. The Main-900 sample deliberately enriched rare and method-discordant cases; its class proportions and unweighted accuracy are therefore not estimates of corpus prevalence. Fourth, disaster stage was only a contextual proxy for the stimulus environment. The study did not measure individual exposure to warnings, media reports, government messages, rescue information, rainfall, or flood levels and thus did not test a complete causal SOR sequence. Fifth, chi-square tests and percentage-point differences are unadjusted and cannot resolve confounding, endogeneity, repeated posting, or reverse direction. The present results should therefore be read as exploratory bivariate associations; the proposed multivariable or multilevel logistic models are a prospective design recommendation, not analyses performed in this study. Future work should use external pre-specified human annotations, probabilistic or calibrated classifiers, multi-platform and survey data, exposure measures, and online-to-offline outcome linkage. Specifically, future research should pre-specify a binary logistic or multilevel model for each behavioral-response outcome and, where valid measures are available, control for disaster stage, regional impact, account type, posting intensity, demographic characteristics, and verified message or hazard exposure, with user-clustered or otherwise robust standard errors.

## Figures and Tables

**Table 1:** Data collection keyword list.

Type	Keywords
Core keywords	North China rainstorm; Haihe River Basin flood; Doksuri; Zhuozhou flood; Zhuozhou inundation
Extended keywords	Inundated; flood discharge; rescue; subway

**Table 2:** Division of disaster event stages.

Stage	Time Period	Basis and Characteristics
Warning stage	27–29 July	Typhoon Doksuri approached and made landfall; meteorological departments issued rainstorm warnings; public attention began to increase.
Outbreak stage	30 July–2 August	Rainfall reached its peak; severe flooding occurred in Zhuozhou and other areas; public attention was high and help-seeking information became concentrated.
Rescue stage	3–8 August	Rescue forces were fully mobilized; water levels gradually receded; discussions shifted toward rescue progress and material support.
Recovery stage	9–15 August	Post-disaster cleanup and recovery began; public discussion shifted toward reflection, accountability, and experience summaries.

**Table 3:** Examples of seed words in the risk perception dictionary.

Perception Dimension	Examples of Seed Words/Phrases	Example Weibo Text
Severity perception	Severe; extreme; historical record; disaster; casualties; heavy losses; shocking	“This rainstorm is too severe; it is simply a disaster.”
Susceptibility perception	Afraid; worried; near my home; will it; please stay safe; pray for safety	“The area downstairs from my home has already been flooded. I am so scared.”
Uncertainty perception	Do not know; unclear; why; exactly; unclear situation; confusing information	“What is the situation now? Does anyone know?”
Trust perception: positive/negative	Trust; stay strong; effective rescue; reassured; concealment; dereliction of duty; no warning	“I trust the government. The rescue workers have worked so hard!”

**Table 4:** Coding scheme for behavioral responses.

Behavior Category	Subcategory	Examples of Keywords/Judgment Rules
Information behavior	Information seeking	“asking”; “what is the current situation?”; “any latest updates?”
	Information sharing/dissemination	Reposting warnings or disaster information; containing words such as “spread” and “please share”
	Following official accounts	Mentions, quotes, or reposts verified government, meteorological, emergency-management, mainstream-media, or rescue-organization accounts as an information source.
Protective behavior	Suggestions/reminders	“do not go out”; “stay away from utility poles”; “prepare some supplies”
	Hazard reporting	“Serious waterlogging on XX Road”; “sandbags are needed here”
Mutual aid behavior	Help-seeking	“help”; “who has a rescue boat?”; “elderly people are trapped”
	Providing help	“I have a place to stay”; “supplies are available”
	Emotional support	“stay strong”; “stay safe”; “hold on”
Expressive behavior	Emotional venting	“This is terrifying”; “I am crying”; “so desperate”
	Questioning and accountability	“Why was work not suspended earlier?”; “What happened to the drainage system?”
	Reflective suggestions	“How should this be improved in the future?”; “Lessons for urban planning”

**Table 5:** Topic labels and core keywords.

Topic	Core Keywords
T1: Property loss and compensation claims	Flood; inundated; loss; compensation claim
T2: Rescue operations	Rescue; rescue team; evacuation; trapped; rushing to aid
T3: Government disaster relief and material support	Disaster relief; flood control; emergency response; donation; materials
T4: Meteorological warnings and disaster reports	Rainstorm; typhoon; Doksuri; red warning
T5: Mutual aid, help-seeking, and disaster records	Mutual aid; trapped; waterlogging; help-seeking
T6: Tributes to heroes and touching stories	Firefighter; sacrifice; hero; Feng Zhen

**Table 6:** Topic intensity distribution across stages (%).

Topic	Warning Stage	Outbreak Stage	Rescue Stage	Recovery Stage
T1: Property loss and compensation claims	2.9	13.9	20.7	20.4
T2: Rescue operations	1.2	18.4	24.8	13.1
T3: Government disaster relief and material support	11.6	14.3	21.2	33.6
T4: Meteorological warnings and disaster reports	82.0	23.2	5.4	19.4
T5: Mutual aid, help-seeking, and disaster records	1.9	24.9	14.5	4.3
T6: Tributes to heroes and touching stories	0.4	5.2	13.4	9.2

**Table 7:** Hit rates of risk perception dimensions across stages (%).

Stage	Severity	Susceptibility	Uncertainty	Trust (Positive)	Trust (Negative)
Warning stage	87.3	15.4	56.4	8.7	1.8
Outbreak stage	61.6	41.2	22.8	26.4	3.1
Rescue stage	51.1	39.4	14.8	42.2	6.9
Recovery stage	53.8	30.6	17.3	37.2	9.3

**Table 8:** Classification performance of the BERT model across risk perception dimensions.

Dimension	Precision	Recall	F1 Score
Severity	0.796	0.923	0.855
Susceptibility	0.826	0.842	0.834
Uncertainty	0.823	0.586	0.685
Trust (positive)	0.812	0.871	0.840
Trust (negative)	0.889	0.333	0.485
Average	0.829	0.711	0.740

**Table 9:** Quantitative comparison between the dictionary-based method and the BERT-based method.

Perception Dimension	Dictionary Hits	BERT Hits	Overlap	Additional Hits by BERT
Severity	17,718	18,689	16,005	2684
Susceptibility	11,930	10,543	9093	1450
Uncertainty	6161	4005	3208	797
Trust (positive)	10,619	10,213	8510	1703
Trust (negative)	1683	260	195	65

**Table 10:** Overall distribution of public behavioral responses.

Behavior Category	Subcategory	Frequency	Proportion Within Subcategory (%)	Proportion Within Category (%)
Information behavior	Information seeking	746	8.5	23.5
	Information sharing	1248	14.2	
	Following official accounts	6758	77.1	
Protective behavior	Suggestions/reminders	4970	53	24.8
	Hazard reporting	4411	47	
Mutual aid behavior	Help-seeking	8391	42.1	43.1
	Providing help	4053	20.3	
	Emotional support	7496	37.6	
Expressive behavior	Emotional venting	1438	45.6	8.6
	Questioning and accountability	304	9.6	
	Reflective suggestions	1408	44.7	

**Table 11:** Hit rates of the four major behavioral responses categories across stages (%).

Stage	Information Behavior	Protective Behavior	Mutual Aid Behavior	Expressive Behavior
Warning stage	69.6	39.3	2.6	9.1
Outbreak stage	27.5	33.0	48.6	8.0
Rescue stage	19.0	22.7	55.8	9.4
Recovery stage	37.8	24.3	24.6	16.8

**Table 12:** Comparison of behavioral hit rates between official accounts and ordinary users (%).

Subcategory	Official Accounts	Ordinary Users
Information seeking	2.3	2.5
Information sharing	3.3	4.8
Following official accounts	24.3	19.0
Suggestions/reminders	19.6	12.0
Hazard reporting	15.2	13.1
Help-seeking	28.1	25.8
Providing help	13.2	12.9
Emotional support	25.5	22.6
Emotional venting	4.0	5.3
Questioning and accountability	0.9	1.1
Reflective suggestions	4.7	4.3

**Table 13:** Unadjusted associations between risk-perception dimensions and behavioral-response indicators.

Behavioral Responses Subcategory	Severity	Susceptibility	Uncertainty	Trust (Positive)	Trust (Negative)
Information seeking	1.3	1.4	3.9	0.8	0.6
Information sharing	3.4	0.5	0.4	1.0	0.2
Following official accounts	8.1	−5.3	**20.0**	1.3	−5.4
Suggestions/reminders	6.5	5.0	10.0	−2.1	6.0
Hazard reporting	**12.2**	9.7	2.5	7.5	8.7
Help-seeking	7.4	**23.2**	0.2	**19.7**	3.2
Providing help	−3.7	−0.4	−9.2	**11.4**	9.1
Emotional support	−9.8	**25.1**	**−16.2**	**47.7**	−4.7
Emotional venting	3.8	3.1	2.0	−1.2	**13.2**
Questioning and accountability	0.9	0.0	1.5	0.4	**16.2**
Reflective suggestions	3.4	2.4	5.3	2.9	**12.3**

Note: All listed associations passed the chi-square test, *p* < 0.05. Bold values indicate the most prominent association pairs, with an absolute difference greater than 10 percentage points. Associations involving negative trust in this table should be interpreted as exploratory textual signals rather than as validated measures of public institutional distrust, given its low positive prevalence, low precision (dictionary = 0.133; BERT = 0.220), and prevalence-sensitive Cohen’s kappa of 0.450 in the Main-900 validation.

**Table 14:** Top three strongest perception–behavior associations across stages.

Rank	Warning Stage	Outbreak Stage	Rescue Stage	Recovery Stage
1	Negative trust → reflective suggestions +62.3% ***	Positive trust → emotional support +33.3% ***	Positive trust → emotional support +55.6% ***	Negative trust → reflective suggestions +30.5% ***
2	Negative trust → hazard reporting +47.6% ***	Negative trust → reflective suggestions +28.7% ***	Susceptibility → emotional support +26.4% ***	Positive trust → emotional support +30.6% ***
3	Positive trust → suggestions/reminders +33.3% ***	Susceptibility → emotional support +24.5% ***	Positive trust → help-seeking +23.0% ***	Negative trust → emotional venting +28.8% ***

Note: *** *p* < 0.001. Percentage-point difference = behavioral rate in the perception group − behavioral rate in the non-perception group. The stage-specific negative trust estimates shown here are exploratory. Their interpretation is constrained by low positive prevalence, low precision in the Main-900 validation (dictionary = 0.133; BERT = 0.220), and a prevalence-sensitive Cohen’s kappa of 0.450; these estimates should not be treated as validated measures of public institutional distrust.

**Table 15:** Regional differences in core perception–behavior associations.

Perception–Behavior Association	Severely Affected Areas	Generally Affected Areas	Unaffected Areas
Positive trust → emotional support	+49.6% ***	+39.1% ***	+29.1% ***
Susceptibility → help-seeking	+21.6% ***	+19.2% ***	+10.9% ***
Severity → hazard reporting	+14.0% ***	+12.6% ***	+3.5% ***
Negative trust → questioning and accountability	+16.4% ***	+6.9% ***	+5.9% ***
Uncertainty → following official accounts	+7.1% ***	+31.1% ***	+32.7% ***
Susceptibility → emotional support	+23.3% ***	+23.9% ***	+23.4% ***

Note: *** *p* < 0.001. Percentage-point difference = behavioral rate in the perception group − behavioral rate in the non-perception group.

**Table 16:** Differences in core perception–behavior associations by user type.

Perception–Behavior Association	Official/Verified Accounts	Ordinary Users	Difference
Positive trust → emotional support	+45.6% ***	+46.4% ***	0.8%
Susceptibility → help-seeking	+23.0% ***	+20.4% ***	2.6%
Uncertainty → following official accounts	+21.8% ***	+16.8% ***	5.0%
Severity → hazard reporting	+13.2% ***	+9.1% ***	4.1%
Negative trust → questioning and accountability	+11.7% ***	+15.5% ***	3.8%
Susceptibility → emotional support	+23.0% ***	+26.6% ***	3.6%

Note: *** *p* < 0.001.

**Table 17:** Stage × region cross-analysis of “susceptibility → help-seeking”.

Region	Warning Stage	Outbreak Stage	Rescue Stage	Recovery Stage
Severely affected areas	+0.0%	+20.1% ***	+21.0% ***	+16.3% ***
Generally affected areas	+9.4% ***	+14.7% ***	+25.2% ***	+2.2%
Unaffected areas	+3.1%	+6.4% ***	+19.3% ***	+3.8% ***

Note: *** *p* < 0.001.

**Table 18:** Stage × region cross-analysis of “positive trust → emotional support”.

Region	Warning Stage	Outbreak Stage	Rescue Stage	Recovery Stage
Severely affected areas	—	+36.9% ***	+57.1% ***	+39.1% ***
Generally affected areas	+2.6%	+19.2% ***	+60.3% ***	+21.8% ***
Unaffected areas	+1.4%	+24.7% ***	+39.6% ***	+22.3% ***

Note: *** *p* < 0.001.

## Data Availability

De-identified derived data, coding resources, and analysis scripts supporting the findings of this study are available from the corresponding author upon reasonable request. Raw Weibo posts are not publicly shared because of platform terms of service and privacy considerations.
